# Toll-Like Receptors Serve as Biomarkers for Early Diagnosis and Prognosis Assessment of Kidney Renal Clear Cell Carcinoma by Influencing the Immune Microenvironment: Comprehensive Bioinformatics Analysis Combined With Experimental Validation

**DOI:** 10.3389/fmolb.2022.832238

**Published:** 2022-01-21

**Authors:** Xiong Zou, Bingqian Guo, Qiang Ling, Zengnan Mo

**Affiliations:** ^1^ Department of Urology, The First Affiliated Hospital of Guangxi Medical University, Nanning, China; ^2^ Center for Genomic and Personalized Medicine, Guangxi Medical University, Nanning, China; ^3^ Guangxi Collaborative Innovation Center for Genomic and Personalized Medicine, Nanning, China; ^4^ Guangxi Key Laboratory for Genomic and Personalized Medicine, Guangxi Key Laboratory of Colleges and Universities, Nanning, China; ^5^ Collaborative Innovation Center of Regenerative Medicine and Medical BioResource Development and Application, Guangxi Medical University, Nanning, China

**Keywords:** KIRC, TLRs, prognosis, diagnosis, biomarkers

## Abstract

**Background:** Toll-like receptors (TLRs) are important initiators of innate and acquired immune responses. However, its role in kidney renal clear cell carcinoma (KIRC) remains unclear.

**Methods:** TLRs and their relationships with KIRC were studied in detail by ONCOMINE, UALCAN, GEPIA, cBioPortal, GeneMANIA, FunRich, LinkedOmics, TIMER and TRRUST. Moreover, we used clinical samples to verify the expressions of TLR3 and TLR4 in early stage of KIRC by real-time fluorescence quantitative polymerase chain reaction (RT-qPCR), flow cytometry (FC) and immunohistochemistry (IHC).

**Results:** The expression levels of TLRs in KIRC were generally different compared with adjacent normal tissues. Moreover, the expressions of TLR3 and TLR4 elevated significantly in the early stage of KIRC. Overexpressions of TLR1, TLR3, TLR4 and TLR8 in KIRC patients were associated with longer overall survival (OS), while inhibition of TLR9 expression was related to longer OS. Additionally, overexpressions of TLR1, TLR3 and TLR4 in KIRC patients were associated with longer disease free survival (DFS). There were general genetic alterations and obvious co-expression correlation of TLRs in KIRC. The PPI network between TLRs was rather complex, and the key gene connecting the TLRs interaction was MYD88. The GO analysis and KEGG pathway analysis indicated that TLRs were closely related to adaptive immunity, innate immunity and other immune-related processes. RELA, NFKB1, IRF8, IRF3 and HIF1A were key transcription factors regulating the expressions of TLRs. What’s more, the expression levels of all TLRs in KIRC were positively correlated with the infiltration levels of dendritic cells, macrophages, neutrophils, B cells, CD4^+^ T cells and CD8^+^ T cells. Finally, the results of RT-qPCR, FC and IHC confirmed that TLR3 and TLR4 were significantly elevated in the early stage of KIRC.

**Conclusion:** The occurrence and development of KIRC are closely related to TLRs, and TLRs have the potential to be early diagnostic biomarkers of KIRC and biomarkers for judging the prognosis and immune status of KIRC. This study may provide new insights into the selection of KIRC immunotherapy targets.

## Introduction

Renal cell carcinoma (RCC) can be divided into different subtypes according to its histological characteristics with unique genetic and molecular alterations, different clinical processes and therapeutic responses (Linehan et al., 2010; Linehan and Ricketts, 2013; Moch et al., 2016). RCC is a tumor originating from renal epithelium (Sanchez-Gastaldo et al., 2017; Zou and Mo, 2021). The most common pathological subtype of RCC is kidney renal clear cell carcinoma (KIRC), accounting for about 75% of RCC (Linehan and Ricketts, 2019). Lipid accumulation and storage are the main pathological features of KIRC (Xiao et al., 2019), and KIRC is the most common cause of death associated with RCC (Hsieh et al., 2017). Local KIRC can be treated by surgical resection, but the treatment of advanced KIRC is still a clinical challenge, and the 5-years overall survival (OS) rate is 0–20% (Petitprez et al., 2021). Patients with recurrent or distant metastasis of KIRC have a poor prognosis and a short median survival (Zheng et al., 2017), moreover, the prognosis of KIRC patients is mainly based on tumor lymph node metastasis (TNM) stage (Pichler et al., 2013; Qin et al., 2013; Zheng et al., 2017), and there is a lack of biomarkers to determine the prognosis of patients, so the identification of early diagnostic biomarkers for KIRC and biomarkers that can assess patient prognosis are critical to the management and treatment of patients with KIRC.

Toll-like receptors (TLRs) are considered to be the key to identify pathogens and control immune response (Marin-Acevedo et al., 2018). TLRs play a crucial role in both innate and subsequent adaptive immunity because of its ability to sense foreign substances, known as pathogen-associated molecular patterns (PAMPs) (Shetab Boushehri and Lamprecht, 2018). In addition to PAMPs, TLRs can also recognize endogenous ligands. When tissue damage or cell death occurs, cells secrete alarmins, also known as danger-associated molecular patterns (DAMPs), but excessive release of the substance is associated with autoimmune diseases and cancer (Chan et al., 2012; Zhao et al., 2014; Urban-Wojciuk et al., 2019). TLRs are expressed in a variety of cells, including immune cells, fibroblasts, and epithelial cells, and their primary role is to protect the host against microbial infection (Kawasaki and Kawai, 2014; Chen et al., 2018). More and more studies have shown that TLRs also play an important role in the occurrence and development of cancer (Wang et al., 2018; Wu et al., 2018), and different TLRs play different roles in different cancers (Dajon et al., 2017). However, the effect of TLRs on KIRC and its mechanism are not clear. In this study, we systematically investigated the expressions of TLRs in KIRC by ONCOMINE, UALCAN and GEPIA databases, and analyzed the relationships between the expressions of TLRs and tumor stage and prognosis in patients with KIRC by GEPIA. In addition, we obtained the genetic alteration information of TLRs and spearman’s correlation of co-expression between TLRs through cBioPortal. We explored the protein-protein interaction (PPI) network of TLRs by GeneMANIA, and obtained the most critical gene associated with TLRs by FunRich, and conducted detailed GO analysis and KEGG pathway analysis of TLRs through LinkedOmics database. At the same time, we investigated the key transcription factors regulating TLRs through TRRUST. What’s more, we studied the relationships between the expression levels of TLRs in KIRC and the levels of immune cell infiltration by TIMER, and evaluated the effects of TLRs and immune cell infiltration on the survival risk of KIRC. Finally, we used clinical samples to verify the expressions of TLR3 and TLR4 in early stage of KIRC by RT-qPCR, flow cytometry (FC) and immunohistochemistry (IHC). Our study provides new insights into TLRs and their relationships with KIRC, contributing to the research of early diagnosis and therapeutic targets of KIRC.

## Materials and Methods

### ONCOMINE

ONCOMINE is a powerful bioinformatics tool for genome-wide expression analysis (Rhodes et al., 2004). The expressions of TLRs in renal cell carcinoma were evaluated by ONCOMINE, so as to speculate the expressions of TLRs in KIRC. In this study, we used the following criteria: *p* value as 0.05, gene rank as top 10%, fold change as 2 and data type as all (DNA and mRNA).

### UALCAN

UALCAN is a website for mining TCGA and MET500 cohort data. It has a variety of functions, including evaluating the expressions of different genes in different cancers and the effect of gene expression on cancer survival (Chandrashekar et al., 2017). Through the “TCGA Gene Analysis” of the UALCAN, this study explored the expressions of TLRs in KIRC compared with normal tissues.

### GEPIA

GEPIA is a website that can be used to analyze RNA expression levels in a variety of tumors and corresponding normal tissues. It also has many functions such as evaluating the effect of different RNA expression levels on the prognosis of cancer (Tang et al., 2017). In this study, we compared the expressions of TLRs mRNA in KIRC and corresponding normal tissues, as well as the expression levels of TLRs in different stage of KIRC through the “Expression DIY” module of GEPIA. Additionally, we also investigated the effects of TLRs on the survival of patients with KIRC through the “Survival” module.

### cBioPortal

cBioPortal is a powerful website for analyzing multidimensional cancer genome data. It can visualize data from cancer tissues and cells into easy-to-understand genetic and gene co-expression events (Gao et al., 2013). Based on the TCGA database of 538 cases of renal clear cell carcinoma (TCGA, Firehose Legacy), the genetic alteration and co-expression of TLRs were obtained from cBioPortal, the threshold of Protein expression z-scores (RPPA) and mRNA expression z scores (RNA Seq V2 RSEM) was set to ±2.0.

### GeneMANIA

GeneMANIA is a convenient and versatile website for analyzing PPI, co-expression, pathways and related functions (Warde-Farley et al., 2010). This study studied the related functions of TLRs and its PPI network through GeneMANIA.

### FunRich

FunRich (3.1.3 exe), a bioinformatics tool, can perform analysis of multiple genes or proteins data sets provided (Fonseka et al., 2020). Through FunRich, we obtained the most critical gene associated with TLRs, and carried out relevant research on this gene.

### LinkedOmics

LinkedOmics is a tool that can be used to analyze the multi-omics data of 32 cancer types from TCGA (Vasaikar et al., 2018). In this study, “GO analysis” and “KEGG Pathway” enrichment analysis of TLRs were carried out using the “Gene Set Enrichment Analysis” tool in “LinkInterpreter” module. We set the “Rank Criteria” as meta *p*-value, the “Minimum Number of Genes Size” as 3 and the “Simulations” as 500. Statistical analysis was conducted using Person Correlation Test.

### TIMER

TIMER, a reliable utility, allows users to enter specific parameters to systematically assess immune cell infiltration in different tumors and its impact on clinical outcomes (Li et al., 2017). In this study, the correlations between the expressions of TLRs and the levels of immune cell infiltration in KIRC were evaluated using the “Gene” module, and the correlations between the clinical prognosis of KIRC and the expressions of TLRs and immune cells were evaluated by “Survival” module.

### TRRUST

TRRUST is a powerful tool for querying transcription factors that regulate gene expression, and can provide regulatory information on the interaction of many transcription factors in humans and mice (Han et al., 2018). In this study, TRRUST was used to query the transcription factors related to regulating TLRs.

### Tissue Collection

Six KIRC tumor tissues and adjacent nontumor tissues were obtained from The First Affiliated Hospital of Guangxi Medical University. The study was approved by the Ethics Committee of The First Affiliated Hospital of Guangxi Medical University (Approval Number: 2021KY-E-182) and all the participants in the experiment gave their informed consent.

### Real-Time Fluorescence Quantitative Polymerase Chain Reaction

Total RNA was extracted by Total RNA Kit I (R6834, Omega). According to the instructions for the use of PrimeScript RT reagent kit (RR036a, Takara, Kyoto, Japan). RNA was reverse transcribed into cDNA, and then cDNA was detected by RT-qPCR using FastStart Essential DNA Green Master (06,924,204,001, Roche) and LightCycler^®^ 96 Instrument (Roche). Three repeated assays were set for each sample. Using glyceraldehyde-3-phosphate dehydrogenase (GADPH) as internal reference, the relative expressions of target genes were analyzed by 2^−ΔΔCt^ method. The primer sequences of TLR3 were as follows: 5′-TTG​CCT​TGT​ATC​TAC​TTT​TGG​GG-3' (Forward); 5′-TCA​ACA​CTG​TTA​TGT​TTG​TGG​GT-3' (Reverse). The primer sequences of TLR4 were as follows: 5′-AGA​CCT​GTC​CCT​GAA​CCC​TAT-3' (Forward); 5′-CGA​TGG​ACT​TCT​AAA​CCA​GCC​A-3' (Reverse).

### Flow Cytometry

Flow cytometry (FC) was used to analyze the expressions of TLR3 and TLR4 in KIRC relative to adjacent nontumor tissues. Fresh human KIRC tissue and adjacent nontumor tissue samples were collected in the First Affiliated Hospital of Guangxi Medical University and prepared into single cell suspension. Firstly, all tissues were cut into small pieces with scissors. After washing twice by D-PBS (311–425-CL, Wisent), the sample was transferred to digestive juice (0.1 mg/ml collagenase I (10,103,578,001, Roche) and 1 mg/ml dnase I (10,104,159,001, Roche) in HBSS (14,025,092, Gibco) solution) and gently shaken at 37°C for 30 min. Digestion was terminated using 10% fetal bovine serum (FBS; SH30070.03, HyClone) in RPMI 1640 and the disaggregated cell suspensions were passed through a 100 μm cell strainer (352,350, Falcon). The cell suspensions were washed thoroughly with D-PBS containing 1% FBS. Red blood cells were eliminated by 1X red blood cell (RBC) lysis buffer (420,301, BioLegend) for 5 min on ice and lysis was terminated by dilution with D-PBS containing 1% FBS, filtered through a 40 μm cell strainer (352,340, Falcon). Then, the cell suspensions were washed thoroughly with D-PBS containing 1% FBS. Finally, cells obtained by centrifugation were resuspended using PBS. Then, single cell suspensions of normal and cancer tissues of kidney were transferred to Fixation Buffer (420,801, Biolegend) for 20 min at room temperature and dark. Precipitation obtained by centrifugation were washed two times by 1× Intracellular Staining Perm Wash Buffer (421,002, Biolegend). Then, about 10^6^ cells were incubated with TLR3 (bs-1444R, Bioss) or TLR4 (bs-20594R, bioss) diluted by 1× Intracellular Staining Perm Wash Buffer for 20 min at room temperature. Cells were washed two times by 1× Intracellular Staining Perm Wash Buffer. The cells were then incubated with Alexa Fluor 488-conjugated donkey anti-rabbit IgG antibodies (1:2000, ab150061, Abcam) for 20 min at room temperature and dark. After washed twice, the cells were resuspended in PBS for flow cytometry analysis. All samples were loaded on a BD C6 Plus for flow cytometry analysis. The data were analyzed using flowjo V10.0.

### Immunohistochemistry

Three cases of KIRC tissues and adjacent nontumor tissues from the First Affiliated Hospital of Guangxi Medical University were collected. After fixation with 4% paraformaldehyde for 24 h, paraffin embedding and section were performed. The sections were dewaxed and hydrated using xylene and gradient alcohol. Then, the sections were treated with EDTA at pH 8.5 (C1034, Solarbio) to induce epitope retrieval by heating. After washing three times with PBS, the sections were incubated with an endogenous peroxidase inhibitor (SP-9001, ZSGB-BIO) for 10 min. Then the sections were washed with PBS three times and incubated with normal goat serum blocking solution (SP-9001, ZSGB-BIO) for 15 min. Primary antibodies (TLR3, 1:200; TLR4, 1:200) were incubated overnight at 4°C. After 30 min of room temperature balance the next day, incubated with Biotinylated Second Antibody (SP-9001, ZSGB-BIO) for 15 min. Then the sections were washed with PBS three times and incubated with Streptavidin-Enzyme Conjugate (SP-9001, ZSGB-BIO) for 15 min. After washing three times with PBS, the sections were incubated with DAB chromogenic fluid (ZLI-9018, ZSGB-BIO) for 5 min. Finally, after redyeing with hematoxylin, the slices were fixed with neutral gum. The images were captured using microscope (Olympus, CX23) and then processed with ImageJ software (NIH).

### Statistical Analysis

All experimental data were statistically analyzed using GraphPad Prism 7. *t*-test was used to analyze the expressions of TLR3 and TLR4 in KIRC tissues relative to adjacent nontumor tissues. In this paper, *p* < 0.05 was considered statistically significant.

## Results

### Toll-Like Receptors Expression Levels in Renal Cell Carcinoma and Adjacent Nontumor Tissues

**FIGURE 1:**
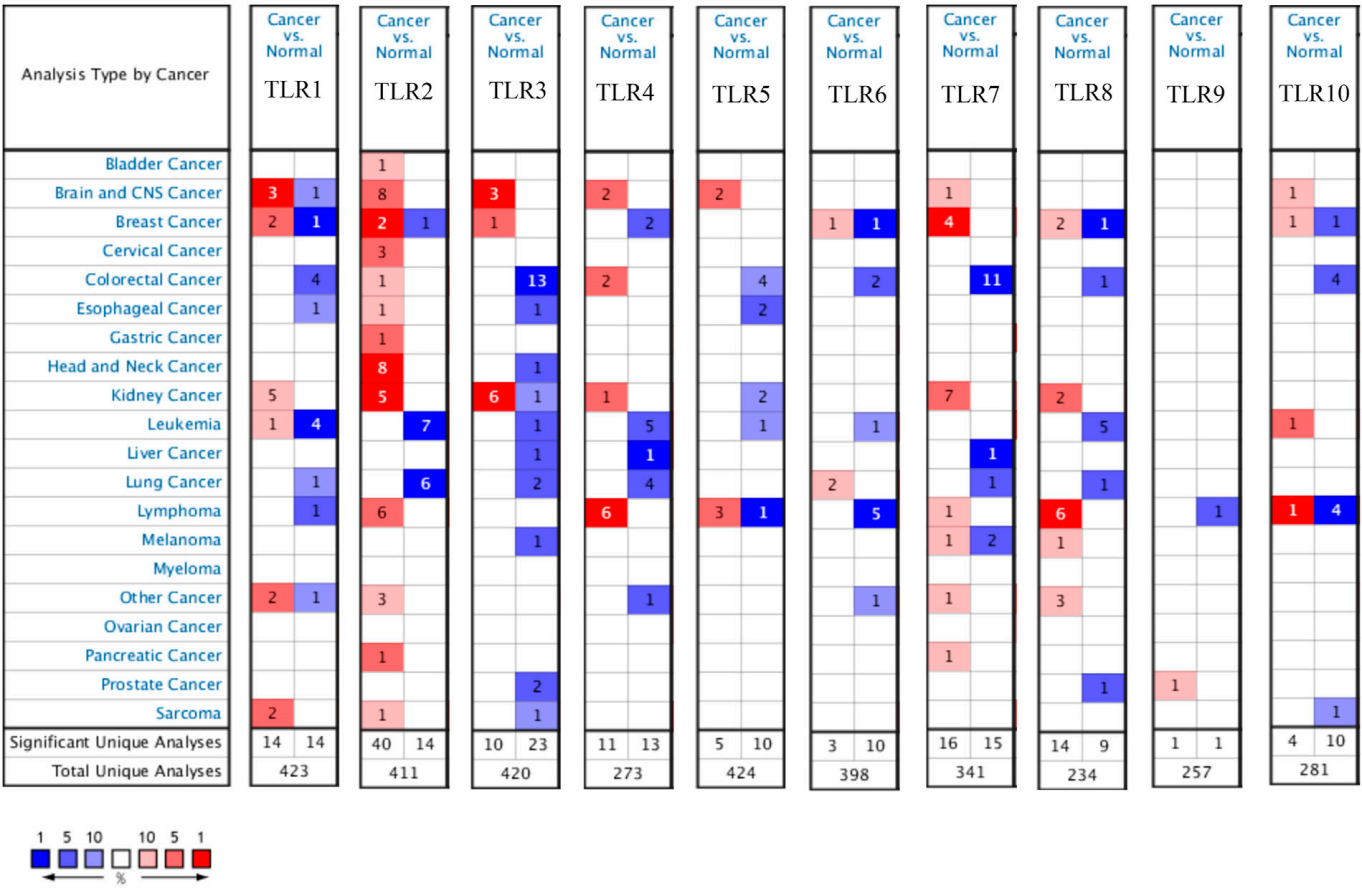
Expression levels of TLRs in kidney cancer. The figure showing expression profiles of TLRs in tumor and paired normal tissue samples from the ONCOMINE database.

**FIGURE 2:**
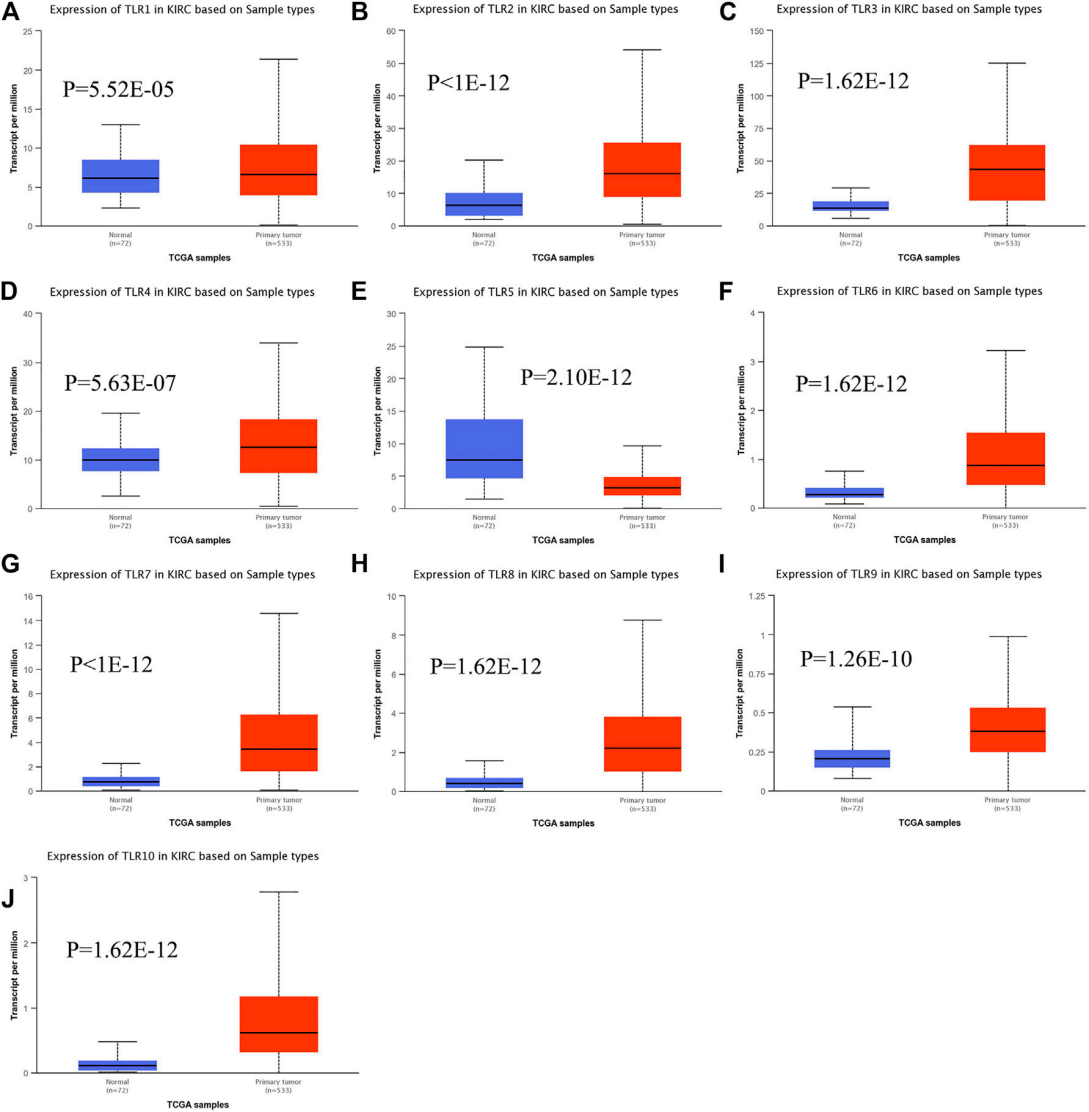
The transcript expression levels of TLRs in KIRC. Box plots showing the transcript expression levels of TLR1 **(A)**, TLR2 **(B)**, TLR3 **(C)**, TLR4 **(D)**, TLR5 **(E)**, TLR6 **(F)**, TLR7 **(G)**, TLR8 **(H)**, TLR9 **(I)** and TLR10 **(J)** in KIRC compared with normal tissues.

**FIGURE 3:**
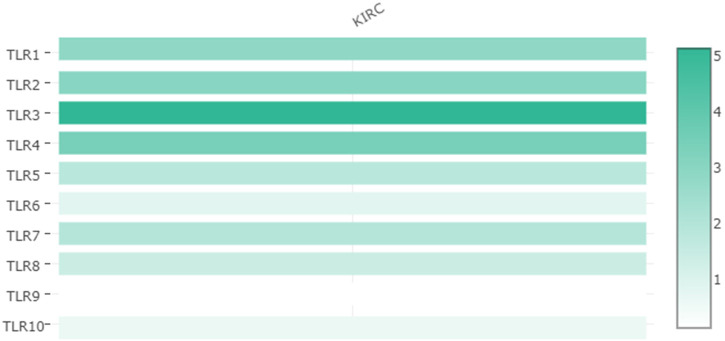
Relative expression levels of TLRs in KIRC.

**FIGURE 4:**
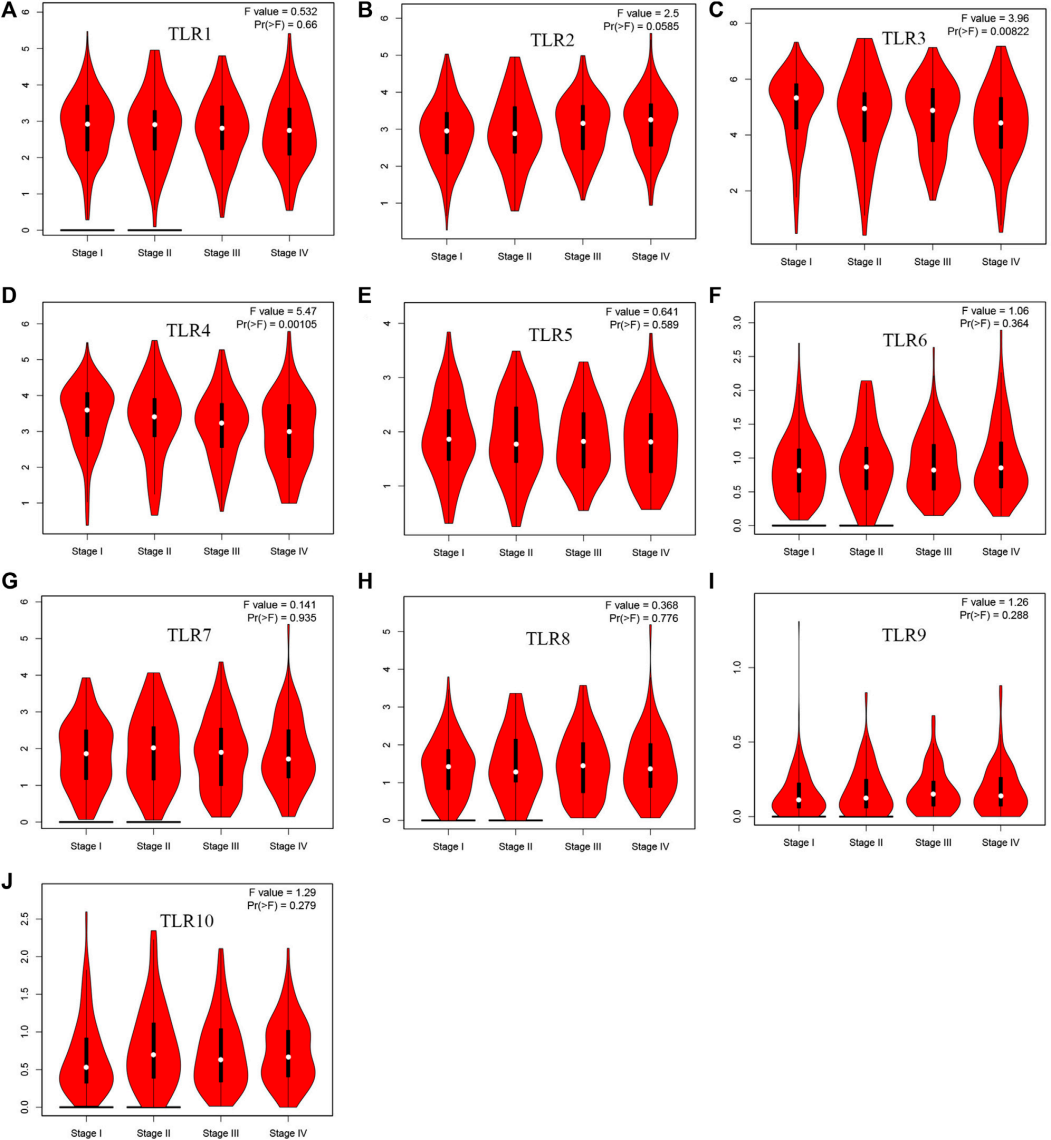
The association of TLRs expression levels with different pathological stages of KIRC. **(A)** TLR1, **(B)** TLR2, **(C)** TLR3, **(D)** TLR4, **(E)** TLR5, **(F)** TLR6, **(G)** TLR7, **(H)** TLR8, **(I)** TLR9, **(J)** TLR10.

Expressions of TLRs in renal cell carcinoma relative to adjacent nontumor tissues were retrieved from ONCOMINE database. The results demonstrated that the expressions of TLR1, TLR2, TLR3, TLR4, TLR7 and TLR8 were significantly elevated, while the expression of TLR5 was significantly decreased in RCC tissues (Figure 1). We also evaluated the transcript expression levels of TLRs in KIRC by UALCAN. Compared with nontumor tissues, the expressions of TLR1 (*p* = 5.52E-05), TLR2 (*p* < 1E-12), TLR3 (*p* = 1.62E-12), TLR4 (*p* = 5.63E-07), TLR6 (*p* = 1.62E-12), TLR7 (*p* < 1E-12), TLR8 (*p* = 1.62E-12), TLR9 (*p* = 1.62E-10) and TLR10 (*p* = 1.62E-12) transcripts in KIRC were significantly elevated, while the transcript expression level of TLR5 was significantly decreased (Figure 2). Meanwhile, we also used GEPIA to compare the relative expression levels of all TLRs in KIRC tissues. The results showed that the TLR3 expression was the highest compared with other TLRs in KIRC tissues (Figure 3). To further identify TLRs associated with the occurrence, progression, and clinical prognosis of KIRC, we evaluated TLRs expression levels at different pathological stages of KIRC. We found that there were significant correlations of TLR3 (*p* = 0.008) and TLR4 (*p* = 0.001) expressions with the pathological stages of KIRC, while there were no significant correlations in the expressions of other TLRs at different pathological stages of KIRC (Figure 4). The expressions of TLR3 and TLR4 elevated significantly in the early stage of KIRC, indicating that TLR3 and TLR4 played an important role in the early diagnosis of KIRC. In addition, all these data indicated that TLRs played a momentous influence in the occurrence and progression of KIRC.

### The Effects of Toll-Like Receptors on the Prognosis of Kidney Renal Clear Cell Carcinoma

**FIGURE 5:**
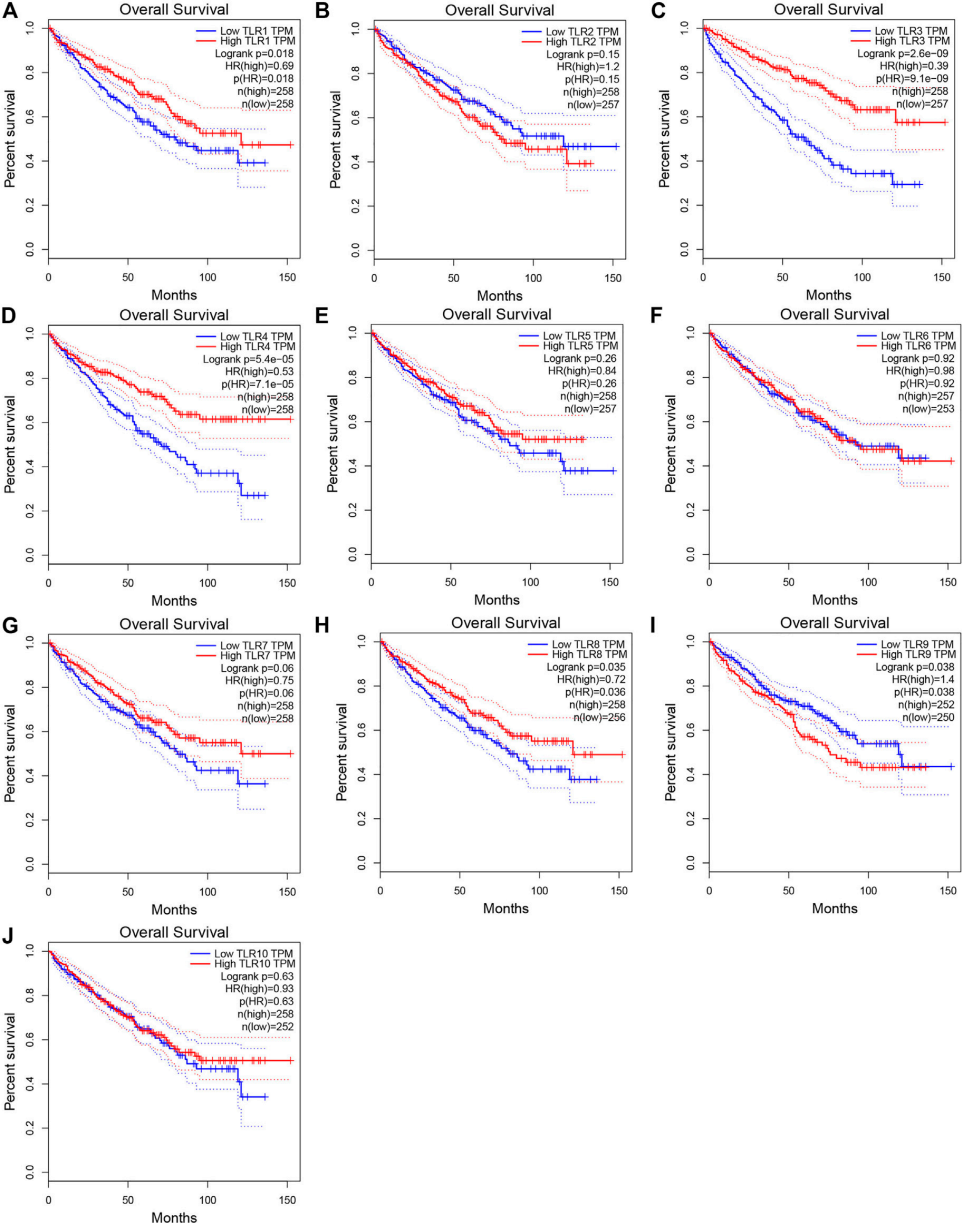
Effects of TLRs on overall survival time of patients with KIRC. **(A)** TLR1, **(B)** TLR2, **(C)** TLR3, **(D)** TLR4, **(E)** TLR5, **(F)** TLR6, **(G)** TLR7, **(H)** TLR8, **(I)** TLR9, **(J)** TLR10.

**FIGURE 6:**
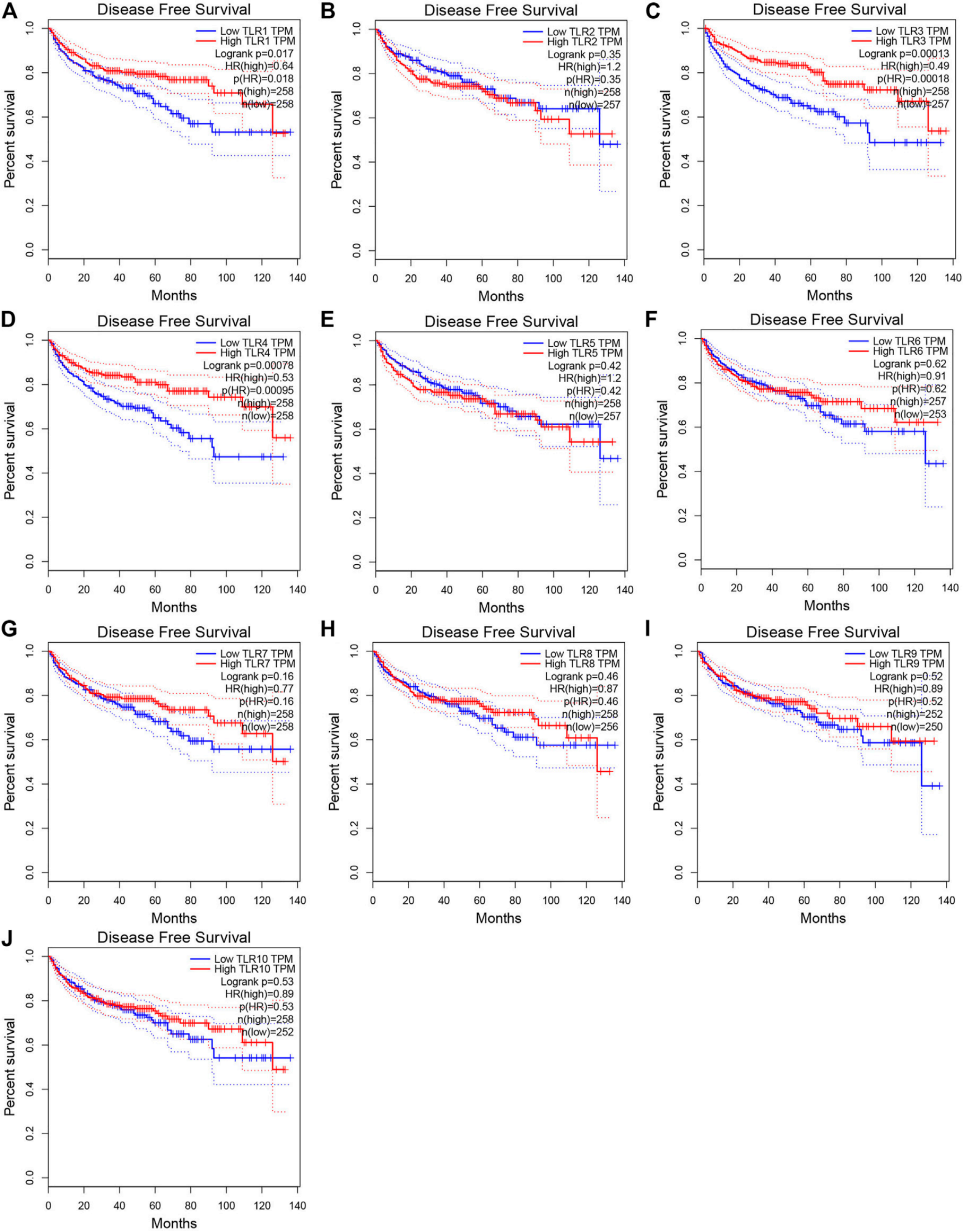
Effects of TLRs on disease free survival time of patients with KIRC. **(A)** TLR1, **(B)** TLR2, **(C)** TLR3, **(D)** TLR4, **(E)** TLR5, **(F)** TLR6, **(G)** TLR7, **(H)** TLR8, **(I)** TLR9, **(J)** TLR10.

In order to evaluate the effects of TLRs on the prognostic value of KIRC, we used GEPIA to assess the correlations of TLRs with the disease free survival (DFS) and overall survival (OS) of KIRC (Figures 5, 6). High expressions of TLR1 (*p* = 0.018), TLR3 (*p* = 2.6e-09), TLR4 (*p* = 5.4e-05) and TLR8 (*p* = 0.035) in patients with KIRC were associated with longer OS (Figures 5A,C,D,H), while low expression of TLR9 (*p* = 0.018) in patients with KIRC was associated with longer OS (Figure 5I). Moreover, we found that high expressions of TLR1 (*p* = 0.017), TLR3 (*p* = 0.00013) and TLR4 (*p* = 0.00078) in patients with KIRC were associated with longer DFS (Figures 6A,C,D), but there were no significant correlations between the expressions of other TLRs and the DFS of KIRC (Figure 6).

### Analyses of Genetic Altetation, Co-Expression and Protein-Protein Interaction of Toll-Like Receptors in Patients With Kidney Renal Clear Cell Carcinoma

**FIGURE 7:**
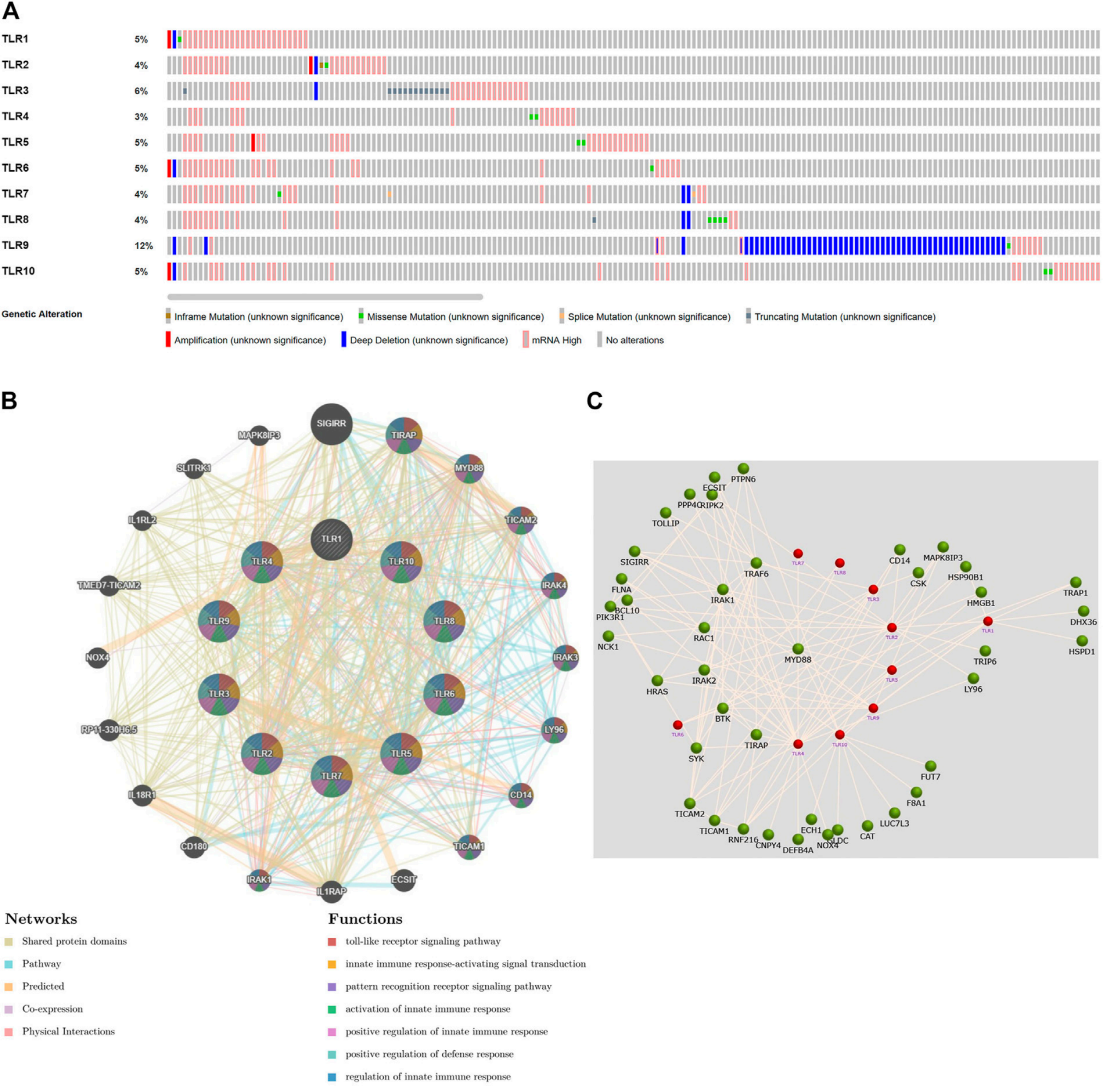
Genetic alteration, function enrichment of PPI network, genetic interaction analyses of TLRs in KIRC patients. **(A)** Genetic alteration of different TLRs in KIRC. **(B)** Functional enrichment of TLRs-related PPI network in KIRC. **(C)** The key gene connecting the TLRs interaction was MYD88.

Next, we systematically analyzed the molecular characteristics of TLRs in patients with KIRC. First of all, we analyzed the genetic alterations and co-expression of TLRs in 538 KIRC patients using cBioPortal. In the KIRC samples, the results showed that the altered/profiled ratio of TLR1, TLR2, TLR3, TLR4, TLR5, TLR6, TLR7, TLR8, TLR9 and TL10 is 5, 4, 6, 3, 5, 5, 4, 4, 12 and 5%, respectively (Figure 7A). High mRNA expression and deep deletion were the most common changes in these samples. Next, we explored the spearman’s correlation of co-expression among TLRs. The results showed that there were general positive correlations between TLRs co-expression (Table 1). Not only that, we also analyzed the PPI network between TLRs through GeneMANIA. The functions of these TLRs were mainly related to toll-like receptor signaling pathway, pattern recognition receptor signaling pathway, innate immune response-activating signal transduction, activation of innate immune response, positive regulation of innate immune response, positive regulation of defense response and regulation of innate immune response (Figure 7B). Next, we further studied the major genes that interacted with TLRs through FunRich. The results showed that MYD88 was the key gene to connect the interaction between TLRs (Figure 7C), indicating that MYD88 plays a crucial role in the expressions of TLRs.

**TABLE 1:** Correlation of co-expression between TLRs.

	TLR1	TLR2	TLR3	TLR4	TLR5	TLR6	TLR7	TLR8	TLR9	TLR10
TLR1	1.000	0.669	0.502	0.613	0.623	0.750	0.870	0.851	0.061	0.678
TLR2	0.669	1.000	0.157	0.352	0.605	0.774	0.679	0.751	0.297	0.661
TLR3	0.502	0.157	1.000	0.467	0.134	0.261	0.441	0.430	−0.268	0.212
TLR4	0.613	0.352	0.467	1.000	0.359	0.497	0.609	0.607	−0.079	0.365
TLR5	0.623	0.605	0.134	0.359	1.000	0.613	0.631	0.605	0.213	0.531
TLR6	0.750	0.774	0.261	0.497	0.613	1.000	0.746	0.792	0.364	0.696
TLR7	0.870	0.679	0.441	0.609	0.631	0.746	1.000	0.901	0.139	0.732
TLR8	0.851	0.751	0.430	0.607	0.605	0.792	0.901	1.000	0.182	0.694
TLR9	0.061	0.297	−0.268	−0.079	0.213	0.364	0.139	0.182	1.000	0.376
TLR10	0.678	0.661	0.212	0.365	0.531	0.696	0.732	0.694	0.376	1.000

### Gene Set Enrichment Analysis of Toll-Like Receptors in Kidney Renal Clear Cell Carcinoma Patients

**FIGURE 8:**
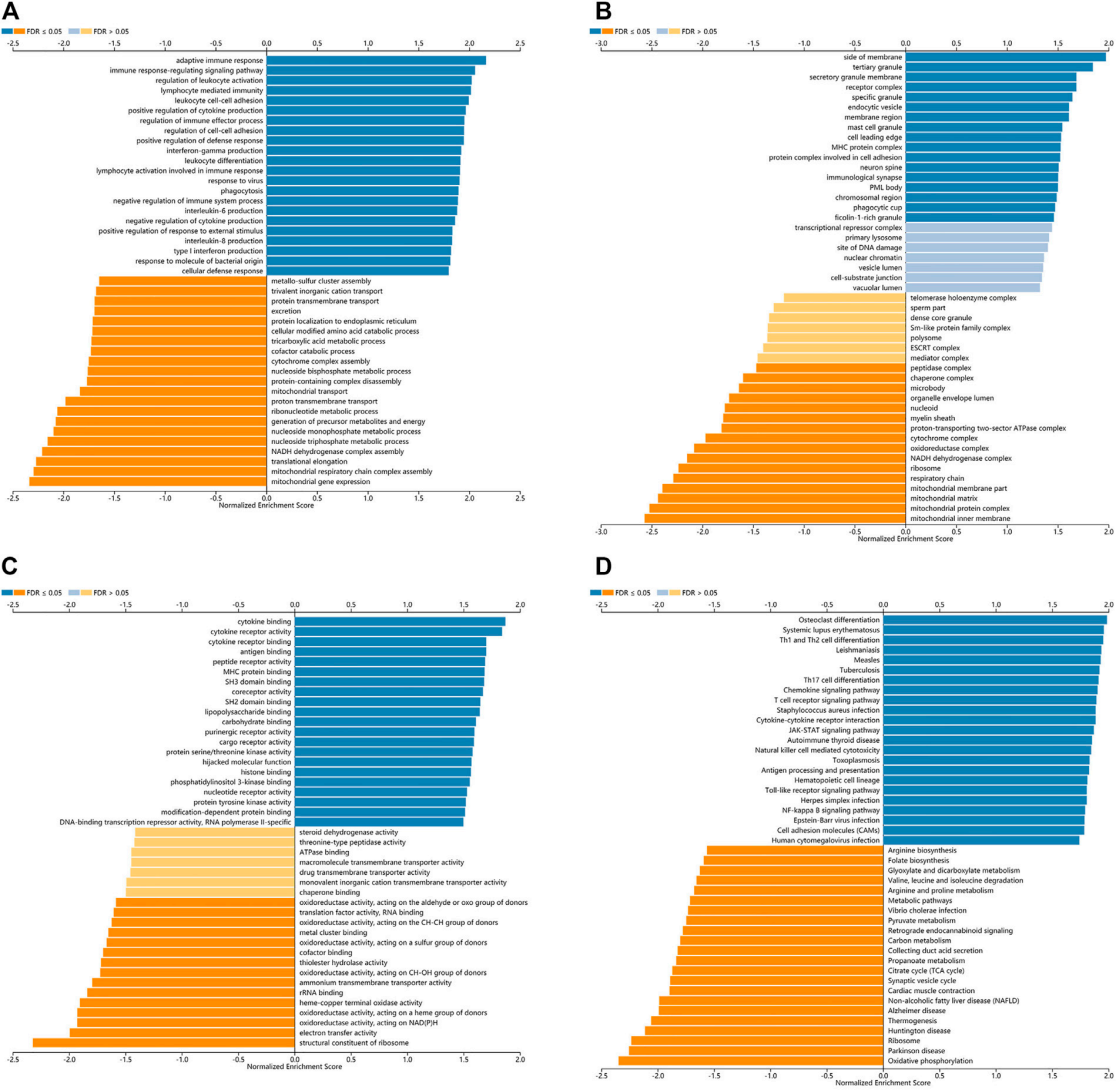
The GO and KEGG pathway analyses of TLRs in KIRC. **(A)** Biological processes involved in TLRs in KIRC. **(B)** Cellular components involved in TLRs in KIRC. **(C)** Molecular functions involved in TLRs in KIRC. **(D)** KEGG pathways involved in TLRs in KIRC.

LinkedOmics was used for gene enrichment analysis of TLRs. We studied TLRs-related GO analysis and KEGG pathway. Many biological processes (BP) of significant enrichment of TLRs were closely related to the occurrence and development of KIRC, including adaptive immune response, regulation of leukocyte activation, immune response-regulating signaling pathway, lymphocyte mediated immunity, leukocyte cell-cell adhesion, positive regulation of cytokine production, interferon-gamma production, regulation of immune effector process, regulation of cell-cell adhesion, positive regulation of defense response, leukocyte differentiation and lymphocyte activation involved in immune response (Figure 8A). In addition, side of membrane, secretory granule membrane, tertiary granule, receptor complex, specific granule, endocytic vesicle, membrane region, mast cell granule, cell leading edge, MHC protein complex, protein complex involved in cell adhesion, neuron spine, immunological synapse, PML body, chromosomal region, phagocytic cup and ficolin-1-rich granule were the most obviously enriched projects in the cellular components (CC) (Figure 8B). The molecular functions (MF) involved in the enrichment of TLRs mainly included cytokine binding, antigen binding, cytokine receptor activity, peptide receptor activity, cytokine receptor binding, MHC protein binding, SH3 domain binding, coreceptor activity, SH2 domain binding, lipopolysaccharide binding, carbohydrate binding and purinergic receptor activity (Figure 8C). Among the TLRs-enriched KEGG pathway, Th17 cell differentiation, Th1 and Th2 cell differentiation, Toll-like receptor signaling pathway, TNF signaling pathway, JAK-STAT signaling pathway and cell adhesion molecules were significantly correlated with the tumorigenesis of KIRC (Figure 8D).

### Key Transcription Factors Associated With Toll-Like Receptors in Kidney Renal Clear Cell Carcinoma Patients

Through TRRUST, we explored the transcription factors that regulated the expression of TLRs in KIRC patients. The results showed that the key transcription factors of TLRs were RELA, NFKB1, IRF8, IRF3 and HIF1A (Table 2). NFKB1 and RELA were key transcription factors that regulated the expressions of TLR2, TLR3, TLR7 and TLR9. IRF3 and IRF8 were key transcription factors that regulated the expressions of TLR3 and TLR4. HIF1A was the key transcription factor that regulated the expressions of TLR2 and TLR6.

**TABLE 2:** The key transcription factors regulating the expressions of TLRs in KIRC patients.

Key TF	Description	Target genes	*p* Value	FDR
RELA	v-rel reticuloendotheliosis viral oncogene homolog A (avian)	4 (TLR2, TLR3, TLR7, TLR9)	1.23E-05	2.31E-05
NFKB1	nuclear factor of kappa light polypeptide gene enhancer in B-cells 1	4 (TLR2, TLR3, TLR7, TLR9)	1.27E-05	2.31E-05
IRF8	interferon regulatory factor 8	2 (TLR3, TLR4)	1.39E-05	2.31E-05
IRF3	interferon regulatory factor 3	2 (TLR3, TLR4)	2.64E-05	3.31E-05
HIF1A	hypoxia inducible factor 1, alpha subunit (basic helix-loop-helix transcription factor)	2 (TLR2, TLR6)	0.00084	0.00084

### Correlations Between the Expressions of Toll-Like Receptors and Immune Cell Infiltration Levels in Kidney Renal Clear Cell Carcinoma Patients

**FIGURE 9:**
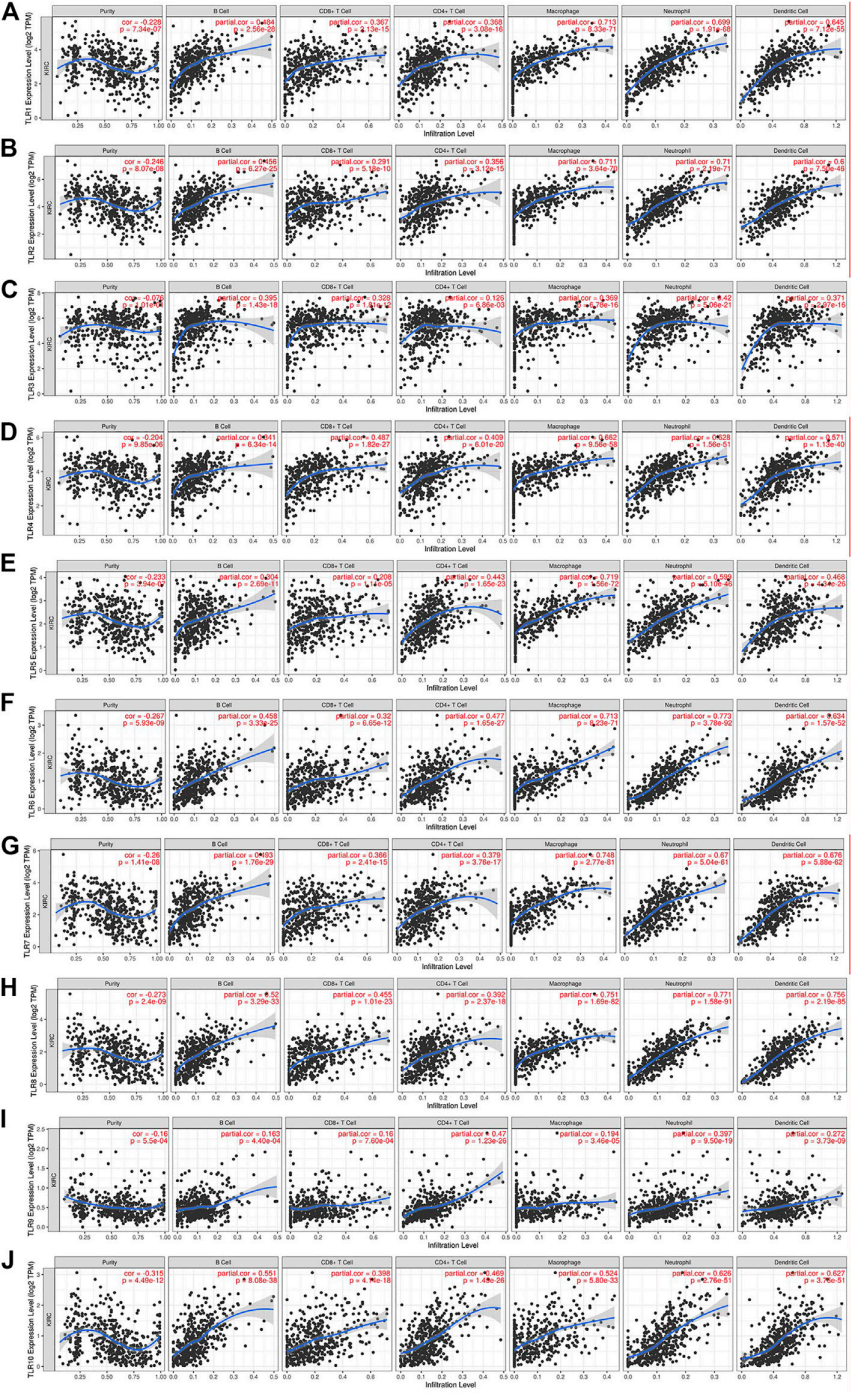
The relationships between the expression levels of TLRs and immune cells infiltration in KIRC. The associations of immune cells infiltration levels with the expression levels of TLR1 **(A)**, TLR2 **(B)**, TLR3 **(C)**, TLR4 **(D)**, TLR5 **(E)**, TLR6 **(F)**, TLR7 **(G)**, TLR8 **(H)**, TLR9 **(I)** and TLR10 **(J)** in KIRC.

We comprehensively studied the correlations between the expressions of TLRs and the levels of immune cell infiltration in patients with KIRC by TIMER. To our surprise, the expressions of all TLRs in KIRC were positively correlated with the infiltration levels of dendritic cells, neutrophils, B cells, macrophages, CD8^+^ T cells and CD4^+^ T cells (Figure 9). Not only that, we also established a cox proportional hazard model of the effects of TLRs and six kinds of immune cells infiltration on patients with KIRC. The results indicated that B cells (coef = −3.497, *p* = 0.031), CD8+T cells (coef = −1.967, *p* = 0.026), CD4+T cells (coef = −4.418, *p* = 0.009), TLR3 (coef = −0.216, *p* = 0.014), TLR4 (coef = −0.394, *p* = 0.024) and TLR8 (coef = −0.926, *p* = 0.002) were negatively associated with the risk of survival in patients with KIRC, while dendritic cells (coef = 4.130, *p* < 0.001) and TLR9 (coef = 1.311, *p* < 0.001) were positively associated with the risk of survival in patients with KIRC (Table 3).

**TABLE 3:** Cox proportional hazard model of TLRs and 6 kinds of immune cells infiltration in KIRC patients.

	Coef	HR	95%CI_l	5%CI_u	p.value	Sig
B_cell	−3.497	0.030	0.001	0.721	0.031	*
CD8_Tcell	−1.967	0.140	0.025	0.788	0.026	*
CD4_Tcell	−4.418	0.012	0.000	0.324	0.009	*
Macrophage	−0.565	0.568	0.029	11.176	0.710	
Neutrophil	2.300	9.977	0.067	1,480.962	0.367	
Dendritic	4.130	62.203	7.655	505.431	<0.001	***
TLR1	0.401	1.494	0.932	2.395	0.096	
TLR2	0.102	1.108	0.848	1.447	0.452	
TLR3	−0.216	0.806	0.679	0.957	0.014	*
TLR4	−0.394	0.674	0.479	0.949	0.024	*
TLR5	−0.082	0.921	0.676	1.254	0.600	
TLR6	0.570	1.768	1.012	3.089	0.045	*
TLR7	0.045	1.046	0.621	1.762	0.867	
TLR8	−0.926	0.396	0.221	0.711	0.002	**
TLR9	1.311	3.709	1.924	7.148	<0.001	***
TLR10	0.077	1.080	0.610	1.913	0.793	

*p < 0.05, **p < 0.01, ***p < 0.001.

### Verification the Expressions of TLR3 and TLR4 in Early Stage of Kidney Renal Clear Cell Carcinoma and Adjacent Nontumor Tissues

**FIGURE 10:**
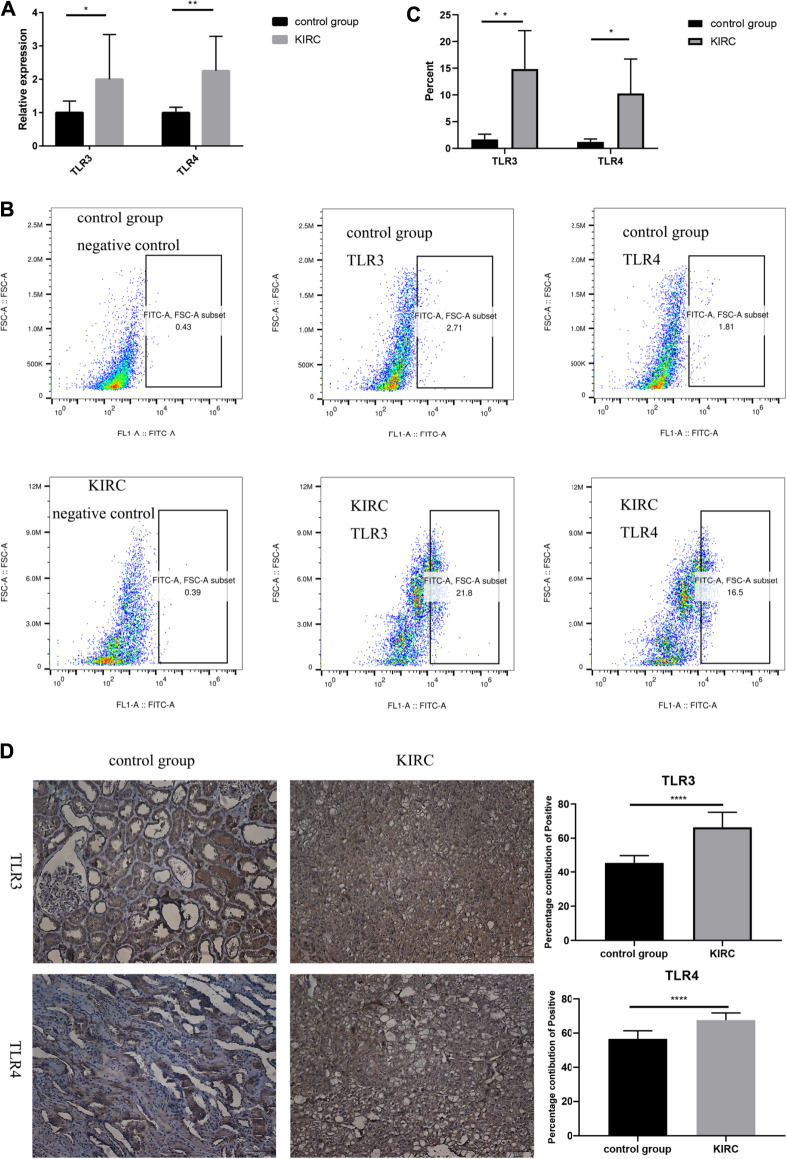
Differential expressions of TLR3 and TLR4 in KIRC and adjacent nontumor tissues. **(A)** Expression levels of TLR3 mRNA and TLR4 mRNA in KIRC relative to adjacent nontumor tissues. **(B, C)** The proportion of TLR3^+^ or TLR4^+^ cells in KIRC and adjacent nontumor tissues was detected by flow cytometry. **(D)** The percentage contribution of TLR3+ or TLR4+ cells in KIRC and adjacent nontumor tissues was detected by IHC. Control group: adjacent nontumor tissues. *p < 0.05; **p < 0.01; ***p < 0.001; ****p < 0.0001.

Finally, we used clinical samples to compare the differences of mRNA and protein expressions of TLR3 and TLR4 in the early stage of KIRC tissues and adjacent nontumor tissues. The characteristics of patients for verifying mRNA and protein expression levels were shown in Tables 4, 5, respectively. The results of RT-qPCR showed that the expression of TLR3 mRNA in KIRC was significantly elevated than that in adjacent nontumor tissues, and TLR4 mRNA also showed the same trend (Figure 10A). What’s more, the results of FC showed that the relative expression of TLR3 at the protein level in KIRC was significantly elevated than that in adjacent nontumor tissues, and the expression of TLR4 at the protein level showed the same trend (Figures 10B,C). In addition, the results of IHC were consistent with the results of RT-qPCR and FC (Figure 10D). Our results confirmed that TLR3 and TLR4 were significantly elevated in the early stage of KIRC compared with adjacent nontumor tissues.

**TABLE 4:** The characteristics of patients for RT-qPCR.

Patients	Sex	Years of age	Tumor location	Tumor size (cm)	TNM stage	Histological type
Sample1	Male	40	Right	4.8 × 4 × 3.5	T_1_N_0_M_0_	KIRC
Sample2	Male	50	Right	2.5 × 2 × 2	T_1_N_0_M_0_	KIRC
Sample3	Female	41	Left	4.5 × 4 × 4	T_1_N_0_M_0_	KIRC

**TABLE 5:** The characteristics of patients for FC.

Patients	Sex	Years of age	Tumor location	Tumor size (cm)	TNM stage	Histological type
Sample4	Male	38	Right	2.5 × 2 × 1.5	T_1_N_0_M_0_	KIRC
Sample5	Male	54	Right	2.5 × 2 × 2	T_1_N_0_M_0_	KIRC
Sample6	Female	64	Left	9 × 9 × 7.5	T_2_N_0_M_0_	KIRC

## Discussion

KIRC can be cured in early diagnosis, but when the disease is metastatic, it is the cancer with the worst prognosis in the urinary system (Angulo et al., 2021). Therefore, early diagnosis of KIRC is important for its therapeutic efficacy and prognosis. However, there is currently a lack of clear clinical biomarkers that can be used to diagnose the early stage of KIRC (Siegel et al., 20182018), and prognosis of patients is mainly determined by TNM stage (Pichler et al., 2013; Qin et al., 2013; Zheng et al., 2017). In addition, molecular biomarkers can provide the possibility of accurate prediction of cancer prognosis and early diagnosis (Tamayo et al., 2011). Therefore, the research on molecular biomarkers for early diagnosis and prognosis of KIRC patients could bring great benefits to the majority of KIRC patients and provide a refined management strategy for KIRC patients.

Toll-like receptor (TLRs) are important initiators of innate and acquired immune responses (Zhang et al., 2021). Ten kinds of TLRs have been identified in humans, and they are expressed in varieties of cells, including B cells, T cells and many other non-immune cells (Nouri et al., 2021; Rameshbabu et al., 2021). There is growing evidence that TLRs play a significance role in a variety of pathological processes, including inflammation, tumor, autoimmune diseases, immunotherapy and vaccination (Vidya et al., 2018). Although the studies of the associations between TLRs and cancer have increased in recent years, there are few studies on the effects of TLRs on KIRC.

Therefore, we used multiple databases to study the relationships between TLRs and KIRC. First of all, we preliminarily studied the expressions of TLRs in kidney cancer through ONCOMINE, and found that the expressions of TLR1, TLR2, TLR3, TLR4, TLR7 and TLR8 in kidney cancer were significantly higher than those in the corresponding normal tissues, while the expression of TLR5 was significantly lower in kidney cancer. Moreover, we further studied the expressions of TLRs in KIRC by UALCAN. The results showed that the expressions of TLRs transcripts in KIRC were elevated than that in adjacent nontumor tissues, except for TLR5. We then evaluated the expressions of all TLRs in KIRC tumor tissues by GEPIA and found that TLR3 was particularly highly expressed in KIRC, followed by TLR4 and TLR2, which was consistent with the results of UALCAN database. Then we further evaluated the expression levels of TLRs at different stages of KIRC, and discovered that the expressions of TLR3 and TLR4 were significantly different at different stages, and TLR3 and TLR4 were significantly increased in the early stage of KIRC. Finally, we also used clinical samples to verify that the expressions of TLR3 and TLR4 were significantly elevated in the early stage of KIRC by RT-qPCR, FC and IHC. These results suggested that TLRs were likely to be important biomarkers for early diagnosis of KIRC, especially TLR3 and TLR4.

Next, we continued to study the effects of TLRs on the survival outcome of KIRC. Overexpressions of TLR1, TLR3, TLR4 and TLR8 significantly prolonged the OS in patients with KIRC, while downregulation of TLR9 significantly prolonged the OS. Moreover, Overexpressions of TLR1, TLR3 and TLR4 significantly prolonged the DFS in patients with KIRC. All of these results suggested that TLRs had the potential to become important biomarkers for predicting prognosis in patients with KIRC, especially TLR1, TLR3, and TLR4.

In order to learn more about TLRs and to understand the possible mechanisms of the effects of TLRs on KIRC patients, we investigated the genetic alteration of TLRs and co-expression of TLRs in KIRC using cBioPortal. There were frequent genetic alterations of TLRs in KIRC. Elevated mRNA expression and deep deletion were the most common changes. Some studies have shown that many factors contribute to the occurrence and development of tumors, and genetic alterations play an indispensable role in this process (Yap et al., 2015; Zeng et al., 2019). What’s more, co-expression of TLRs was found a clear association, suggesting that all of these TLRs play a momentous synergistic role in the occurrence and development of KIRC.

Next, we concentrated on the PPI network, GO analysis and KEGG pathway analysis of TLRs. Not surprisingly, the functions of these TLRs were mainly related to toll-like receptor signaling pathway, activation of innate immune response, pattern recognition receptor signaling pathway, positive regulation of innate immune response, innate immune response-activating signal transduction, positive regulation of defense response and regulation of innate immune response. The GO and KEGG pathway analyses of TLRs indicated that TLRs were mainly associated with regulation of leukocyte activation, immune response-regulating signaling pathway, adaptive immune response, lymphocyte mediated immunity, leukocyte cell-cell adhesion, positive regulation of cytokine production, interferon-gamma production, regulation of immune effector process, regulation of cell-cell adhesion, cytokine binding, positive regulation of defense response, MHC protein complex, protein complex involved in cell adhesion, cytokine receptor activity, cytokine receptor binding, antigen binding, TNF signaling pathway, Th1 and Th2 cell differentiation, Toll-like receptor signaling pathway, Th17 cell differentiation, cell adhesion molecules and JAK-STAT signaling pathway. Some studies have demonstrated that tumorigenesis is closely related to immune dysfunction (Raja et al., 2018; Nakamura et al., 2020). Moreover, our study indicated that TLRs were closely related to adaptive immunity, innate immunity and other immune-related processes, and that genetic alterations in TLRs were very common in KIRC, so we have every reason to believe that the occurrence of KIRC is closely related to TLRs.

Next, through FunRich, we found that the most critical gene that affected the interaction between TLRs was MYD88. Most TLRs depend on MYD88 for the regulation of multiple signal pathways and immune responses (Kim et al., 2019). MyD88 is involved in the development of various cancers by acting downstream of TLRs (Skorka et al., 2021). The results of our study and previous conclusions suggested that MYD88 played a bridging role in human immune homeostasis mediated by TLRs.

In order to learn more about TLRs-related information, we have also explored TLRs-related transcription factors. Our study found that the key transcription factors of TLRs were RELA, NFKB1, IRF8, IRF3 and HIF1A. Previous studies demonstrated that RELA phosphorylation involved in the progression of various diseases including inflammatory disease and cancer by regulating NF-κB signaling (Lu and Yarbrough, 2015) and RELA also played a key role in mediating oncogene-induced aging (Lesina et al., 2016). NFKB1 is a cancer and inflammation inhibitor that plays an inhibitory role in the occurrence and development of a number of cancers by inhibiting the NF-κB signaling pathway (Cartwright et al., 2016; Concetti and Wilson, 2018). In addition, studies have shown that the loss of NFKB1 can lead to inflammation and the progression of cancer by increasing the expression of TNF (Low et al., 2020). IRF8, a tumor suppressor, is also a potential therapeutic option to overcome tumor drug resistance (Wu et al., 2020). IRF3, interferon regulatory factor 3, a tumor suppressor, plays an important role in inhibiting infection and cancer (King et al., 2017; Tian et al., 2020). HIF1A is a hypoxia inducible factor, and its absence increases tumor aggressiveness and metastatic activity (Tiwari et al., 2020). All these results provide insight into the complicated relationship among KIRC, TLRs and transcription factors. In addition, it also provides a further basis for TLRs to become early diagnostic biomarkers and judge the prognosis of patients with KIRC.

The tumor promoting or anticancer effects of TLRs may be related to the tumor microenvironment of immune cell infiltration and the types of cancers (Patra et al., 2020), therefore, we investigated the relationship between the expressions of TLRs in KIRC and the levels of immune cells infiltration by TIMER. Surprisingly, our results showed that the expressions of all TLRs in KIRC were significant positively correlated with the infiltration levels of dendritic cells, macrophages, neutrophils, B cells, CD8^+^ T cells and CD4^+^ T cells. There is growing evidence that immune cells infiltration is an important determinant of tumor therapeutic response and clinical outcome (Bindea et al., 2013; Liu et al., 2017). These results, combined with the differential expression of TLRs in KIRC and the significant effects of TLRs expressions on the prognosis of patients with KIRC, indicate that TLRs have the potential to be early diagnostic biomarkers of KIRC and biomarkers for judging the prognosis and immune status of KIRC patients. Further studies are needed to verify our results and explore how TLRs affect the immune microenvironment of KIRC.

## Conclusion

In summary, the expression levels of TLRs in KIRC were generally different compared with adjacent normal tissues. Moreover, the expressions of TLR3 and TLR4 elevated significantly in the early stage of KIRC. Different TLRs had different effects on the prognosis of KIRC patients. TLRs can be used as important biomarkers for early diagnosis and prognosis assessment in patients with KIRC, especially TLR3 and TLR4. There were general genetic alterations and obvious co-expression correlation of TLRs in KIRC. The PPI network between TLRs was rather complex, and the key gene connecting the TLRs interaction was MYD88. The GO analysis and KEGG pathway analysis indicated that TLRs were closely related to adaptive immunity, innate immunity and other immune-related processes. RELA, NFKB1, IRF8, IRF3 and HIF1A were key transcription factors regulating the expressions of TLRs. What’s more, the expressions of all TLRs in KIRC were significantly positively correlated with the infiltration levels of dendritic cells, macrophages, neutrophils, B cells, CD8^+^ T cells and CD4^+^ T cells. Taken together, the occurrence and development of KIRC is closely related to TLRs, and TLRs have the potential to be early diagnostic biomarkers of KIRC and biomarkers for judging the prognosis and immune status of KIRC patients. The results of our study may provide new insights into the selection of KIRC immunotherapy targets.

## Data Availability

The original contributions presented in the study are included in the article/Supplementary Material, further inquiries can be directed to the corresponding author.
